# Dehydration stress and Mayaro virus vector competence in *Aedes aegypti*

**DOI:** 10.1128/jvi.00695-23

**Published:** 2023-12-05

**Authors:** Jaime Manzano-Alvarez, Gerard Terradas, Christopher J. Holmes, Joshua B. Benoit, Jason L. Rasgon

**Affiliations:** 1 Department of Entomology, The Pennsylvania State University, University Park, Pennsylvania, USA; 2 The Center for Infectious Disease Dynamics, The Pennsylvania State University, University Park, Pennsylvania, USA; 3 Universidad El Bosque, Vicerrectoría de Investigación, Saneamiento Ecológico, Salud y Medio Ambiente, Bogotá, Colombia; 4 The Huck Institutes of the Life Sciences, The Pennsylvania State University, University Park, Pennsylvania, USA; 5 Department of Biological Sciences, University of Cincinnati, Cincinnati, Ohio, USA; University of North Carolina at Chapel Hill, Chapel Hill, North Carolina, USA

**Keywords:** alphavirus, relative humidity, vector competence, vector-borne diseases

## Abstract

The mosquito *Aedes aegypti* is a competent vector of multiple pathogens, including dengue, Zika, yellow fever, chikungunya, and Mayaro viruses. *Ae. aegypti* is highly invasive and is currently present in the Americas, Oceania, Asia, and Europe, but its distribution and the pathogens it transmits are expected to change due to climate change. Relative humidity (RH) is an environmental variable that affects mosquito biology and distribution and can differ between location, habitat, and season, with mosquitoes facing significant variation in RH during their lifespan. Low relative humidity can induce dehydration in mosquitoes, leading to alterations in physiological and behavioral responses relevant to pathogen transmission, such as blood-feeding and host-seeking behavior. In this study, we evaluated the short- and long-term effects of low RH shock on mortality and Mayaro virus infection and vector competence in *Ae. aegypti*. Our results show that exposure to dehydration does not impact viral titers, nor infection, dissemination, and transmission rates in mosquitoes infected with Mayaro virus. However, we detected a significant effect of humidity shock on mosquito mortality and blood-feeding frequency, regardless of infection status. The previously observed effects of higher feeding during dehydration and the current observation of altered survival, along with no impact on vector competence, suggest that the impact of dehydration on viral transmission in mosquitoes will likely be complex.

**IMPORTANCE:**

Relative humidity (RH) is an environmental variable that affects mosquito physiology and can impact pathogen transmission. Low RH can induce dehydration in mosquitoes, leading to alterations in physiological and behavioral responses such as blood-feeding and host‐seeking behavior. We evaluated the effects of a temporal drop in RH (RH shock) on mortality and Mayaro virus vector competence in *Ae. aegypti*. While dehydration induced by humidity shock did not impact virus infection, we detected a significant effect of dehydration on mosquito mortality and blood-feeding frequency, which could significantly impact transmission dynamics.

## INTRODUCTION

Vector-borne diseases (VBDs) are responsible for more than 17% of all reported infectious disease cases and cause ~700,000 deaths worldwide (1). The mosquito *Aedes aegypti*, originally endemic to Africa, is now present worldwide and is a competent vector of many viruses, including dengue, Zika, chikungunya, Mayaro, and yellow fever viruses [reviewed in reference (2)]. Dengue alone accounts for more than 2.3 million reported cases and over 1000 deaths in the Americas in the years 2013, 2015, 2016, 2019, 2020, and 2022 (3, 4). Although this mosquito species represents a significant public health threat, the association between climate and the pathogens that it transmits still requires further investigation (5).

Climatic factors such as precipitation, relative humidity (RH), and temperature affect the distribution of mosquitoes and the pathogens they carry, and these variables are widely used for modeling VBD dynamics (6, 7). For example, decreases in RH induce dehydration stress in the mosquito that alters its physiology and behavior, resulting in reductions in survival, nutrient reserves, oviposition, and egg counts (8). Dehydration in mosquitoes can occur rather quickly; it has been shown that mosquitoes can lose about 20% of their water content in less than 10 hours depending on temperature, RH, and access to water (7). Modeling studies suggest that environmental humidity is a driver of VBD occurrence due to the negative effect that dehydration in mosquitoes has on vectorial capacity (7, 9). Nevertheless, we still require more empirical research to understand the effect of dehydration on vector-pathogen interactions.

RH has been found to be one of the determinants of *Ae. aegypti* distribution because the population of this species fluctuates depending on RH, along with precipitation and temperature (10–13). The current distribution of *Ae. aegypti* is already the widest ever recorded, and it is expected to further expand due to climate change (14, 15). RH is a fluctuating variable that can vary during the day; it has been reported under semi-field conditions that mosquitoes face RHs ranging from 50% to 100%, depending on the time of the day (16). In addition, RH differs between indoor and outdoor settings (17, 18). Weather abnormalities can alter the environmental RH, as is the case for dry heatwaves, which are periods of unusually hot weather characterized by an increase in temperature and a decrease in RH (19). Heatwaves have economic and environmental impacts worldwide, and their frequency is expected to increase due to climate change (20, 21). It is therefore expected that mosquitoes will face variable environmental RH during their lifespan, which is expected to have an impact on the survival of mosquitoes (22).

When RH decreases, mosquitoes invest energy in maintaining their osmotic balance to avoid dehydration through manipulation of their transpiration and evaporation rates or must respond to the physiological impact of water loss (7, 22, 23). Mosquitoes seek out beneficial microclimates, such as shrubs, to decrease water loss (24) and maintain osmotic balance by minimizing water and ion loss through excretion using their highly efficient excretory system (23). When continually exposed to arid conditions, they are able to change their cuticle composition and thickness (25, 26) and can induce morphological changes in their spiracles to avoid further water loss during severe dry seasons (27). In cases where they fail to maintain their osmotic balance over time, mosquitoes become more active and increase their host-seeking and blood-feeding behaviors in an attempt to get needed water before dying of dehydration (7). If dehydration reaches critical levels, specific molecular changes occur to prevent and repair excessive damage, which includes the expression of antioxidants and heat shock proteins (7, 22, 28).

Vector competence is the ability of a vector to become infected with a specific pathogen and transmit it to the next naive host during feeding (2). Environmental stressors, such as changes in temperature, have been previously shown to alter vector competence for alphaviruses and flaviviruses in *Ae. aegypti* (29–31). Because temperature and RH are closely related, we hypothesized that dehydration induced by different treatments of RH shock would affect the viral vector competence of *Ae. aegypti* (vector) for the Mayaro virus (MAYV; -L strain, pathogen). We show that exposure to RH stress affects mosquito mortality and blood-feeding behavior, but we did not observe an effect on viral loads, nor infection, dissemination, or transmission rates (IR, DR, or TR), nor transmission efficiency (TE) of the virus in the mosquitoes.

## MATERIALS AND METHODS

### Mosquito rearing

*Aedes aegypti* Liverpool strain mosquito eggs were originally provided by the NIH/NIAID Filariasis Research Reagent Resource Center for distribution by BEI Resources, NR-48921, NIAID, NIH. Insects were maintained and reared at the Penn State Millennium Sciences Complex insectary (University Park, USA) in 30 × 30 × 30 cm cages, under 27°C ± 1°C, a 12:12 hours light:dark cycle, and 80% RH. Larvae were fed koi pellets (Tetra Pond Koi Vibrance; Tetra, Melle, Germany). Adult mosquitoes were maintained with a 10% sucrose solution *ad libitum*. For reproduction purposes, adult females were allowed to feed on anonymous human blood (BioIVT, NY, USA) following a previously described membrane feeder protocol (32).

### Cells and virus stock

Vero cells (African green monkey kidney origin; CCL-81, ATCC, Manassas, VA, USA) were maintained in complete growth medium [Dulbecco’s modified Eagle’s medium (DMEM) supplemented with 10% fetal bovine serum (FBS) and 1% penicillin and streptomycin] in a 37°C incubator with 5% CO_2_ [all reagents were purchased from Gibco, Thermo Fisher Scientific (Waltham, MA, USA)]. Mayaro virus genotype L strain BeAr505411 (BEI Resources, Manassas, VA, USA) was originally isolated from *Haemagogus janthinomys* mosquitoes in Para, Brazil, in 1991. The virus was diluted in DMEM (multiplicity of infection of 0.01) and propagated in Vero cells for 1 hour, and then cells were washed with DMEM and incubated with 30 mL of complete growth medium for 24 hours. Then, infectious supernatants were aliquoted and stored at –80°C. Prior to their experimental use, viral titers of frozen stock aliquots were measured with focus-forming assays (FFA).

### Humidity treatment setup

Three humidity treatments were prepared at a constant temperature of 27°C: 75% RH, 35% RH, and 80% RH (control), which corresponds to regular insectary humidity conditions. To reach 75% RH and 35% RH conditions, chambers were crafted with plastic transparent containers and cups holding supersaturated solutions of NaCl and MgCl_2_, respectively (33). RH was monitored before and during the experiment using two digital hygrometers (ThermoPro, Ontario, Canada) per chamber.

### Experiment A: Viral infection after exposure to dehydrating conditions

**FIG 1:**
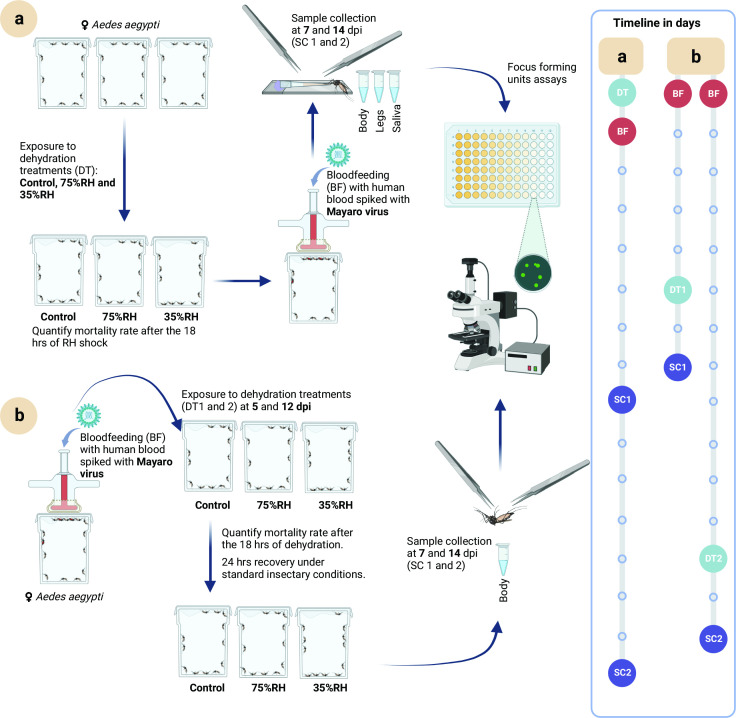
Pipeline for methods used in vector competence study. (a) Long-term effect of 18 hours of exposure (HE) to dehydrating conditions (dehydration treatments). (b) Short-term effect of 18 HE on dehydrating conditions. Mosquitoes did not have access to water nor sugar solution during varying RH exposure. The number of dead mosquitoes was counted just after the 18 HE. Once the exposure time was over, mosquitoes were put under standard insectary conditions (80% RH) with access to a 10% sugar solution (recovery conditions). Infectious blood-feeding and focus-forming assays were performed as in references (32, 32). DT, dehydration treatments; BF, bloodfeeding; SC, sample collection.

Three-to-five-day-old female mosquitoes were anesthetized with ice and sorted into three 20 × 30 × 20 cages in groups of 120, then held for a day at normal insectary conditions to allow them to recover. The next day, mosquitoes were deprived of access to water, and cages were equally divided between the three humidity treatments for 18 hours of exposure (HE); the number of dead mosquitoes per treatment was recorded at the end to calculate the mortality rate. Post-humidity shock, mosquitoes were fed for 1 hour on infected human blood spiked with infectious MAYV (1 × 10^7^ ffu/mL) at regular insectary conditions (27°C and 80% RH). An aliquot of infectious blood was collected, centrifuged, and stored at −80°C for further titration via FFA. Fully engorged mosquitoes anesthetized on ice were sorted, placed in 10 × 10 × 10 cages, and kept at 80% RH with a 10% sucrose solution *ad libitum* for the rest of the experiment. Blood-feeding rate and daily survival were recorded. Vector competence assays (see below) were performed on 20 of the surviving mosquitoes per treatment at 7 and 14 dpi (a total of 120 mosquitoes per replicate). The experiment was run in three biological replicates (Fig. 1a), and the results are reported as the combination of them.

### Experiment B: Viral infection before exposure to dehydrating conditions

Five- to eight-day-old female mosquitoes were anesthetized on ice and sorted into a 20 × 30 × 20 board cage containing 320 females, which were held for a day in regular insectary conditions (27°C and 80% RH). Mosquitoes were then fed for 1 hour on infected human blood spiked with infectious MAYV (1 × 10^7^ ffu/mL). An aliquot of infectious blood was collected, centrifuged, and stored at −80°C for further titration via FFA (see below). Fully engorged mosquitoes anesthetized on ice were sorted and placed in six 10 × 10 × 10 cm cages in groups of 40 and kept at regular insectary conditions. Three cages were randomly selected at 5 dpi and the other three at 12 dpi (for 7 and 14 dpi vector competence, respectively). Selected boxes were then placed separately at the different humidity treatments for 18 HE. Mosquito mortality was assessed before and after exposure to these humidity treatments. After 18 HE, mosquitoes were moved to recovery conditions for 24 hours to allow virus replication. The next day, corresponding to 7 and 14 dpi, viral titers were assayed in up to 20 of the surviving mosquitoes per treatment (first replicate at 7 dpi: 20, 20, and 20; second replicate: 20, 18, and 13; first replicate at 14 dpi: 20, 20, and 16; second replicate: 16, 20, and 12). Whole mosquitoes were individually placed in 300 µL of mosquito dilutant [20% heat-inactivated FBS in Dulbecco’s phosphate-buffered saline (PBS), 50 µg/mL penicillin/streptomycin, 50 µg/mL gentamicin, and 2.5 µg/mL amphotericin B]. Samples were homogenized by a single zinc-plated steel, 4.5 mm bead (Daisy, Rogers, AR, USA) using a TissueLyser II (QIAGEN GmbH, Hilden, Germany) on 30 Hz for 2 min cycle. Samples were clarified by centrifugation and stored at −80°C until FFAs (see below). The experiment was run in two biological replicates (Fig. 1b), and the results are reported as a combination of them.

### Vector competence assays

For Experiment A, at 7 and 14 days post-infection (dpi), mosquitoes were anesthetized with triethylamine (Sigma-Aldrich, St. Louis, MO, USA). Legs were detached from the body, and mosquitoes were forced to salivate into a pipette tip with a 1:1 mix of 50% sugar solution and FBS. Legs, body, and saliva samples were collected in 2 mL safe-lock tubes (Eppendorf, Hamburg, Germany) with 300, 300, and 100 µL of mosquito dilutant (see above), respectively, and placed on ice. Samples from the body and legs were homogenized by a single zinc-plated steel 4.5 mm bead (Daisy, Rogers, AR, USA) using a TissueLyser II (QIAGEN GmbH, Hilden, Germany) on 30 Hz for 2 min cycle. Finally, samples were quickly centrifuged and stored at −80°C for further titration. Body, leg, and saliva samples were used to prepare 10-fold dilutions (10^2^ to 10^5^, 10^1^ to 10^4^, and 10^0^ to 10^1^, respectively) for the FFAs. Vector competence rates were reported as infection rate (IR) that stands for the proportion of infected bodies over the total, dissemination rate (DR) that stands for the proportion of infected legs over infected bodies, transmission rate (TR) that stands for the proportion of infected saliva over infected legs, and transmission efficiency (TE) that stands for the proportion of infected saliva over the total collected mosquitoes.

### Focus-forming assays

The detection of infectious MAYV particles in samples from mosquito bodies, legs, and saliva was carried out by FFAs in Vero cells. Vero cells were seeded in flat 96-well plates at a density of 4 × 10^4^ cells/well. The next day, a series of 10-fold dilutions of the samples were prepared in FBS-free DMEM, and 30 µL was used to infect the cells at 37°C and 5% CO_2_ for 1 hour. Then, supernatants were removed, replaced by 100 µL of overlay medium (1:1 mix of 1.6% methyl cellulose and complete growth medium), and incubated at 37°C and 5% CO_2_. The next day, cells were fixed with 4% paraformaldehyde for 15 min (Sigma, St. Louis, MO, USA), washed thoroughly with PBS, permeabilized with 0.2% Triton X in PBS for another 15 min, and washed with PBS. Viral antigens were detected using the primary monoclonal anti-chikungunya virus E2 envelope glycoprotein clone CHK-48 (BEI Resources, Manassas, VA, USA) (which cross-reacts with MAYV) diluted 1:500 in 1× PBS solution, as previously described (34). Samples were washed with PBS and then treated with the secondary antibody Alexa-488 goat anti-mouse IgG (Invitrogen, Eugene, OR, USA) at a 1:700 dilution with 1× PBS, followed by a last wash with PBS. An Olympus BX41 inverted microscope equipped with an UPlanFI 4× objective and a FITC filter was used for counting MAYV foci.

### Statistical analysis and figure generation

Data were analyzed using R Studio (2023.3.0.386; PBC, Boston, MA, USA). Differences in IR, DR, TR, TE, and mortality, and blood-feeding rates were analyzed using Fisher’s exact test followed by multiple Bonferroni-corrected comparisons. Since our data did not follow a normal distribution, the Kruskal-Wallis test was used to compare viral titers in the body, legs, and saliva; the test was also used to assess differences among replicates. Survival curves were analyzed using a log-rank test, which accounts for censored data. All *P*-values that were below 0.05 (*P* < 0.05) were considered significant. Graphs and plots were made with R Studio (2023.3.0.386; PBC, Boston, MA, USA) and Biorender.com. Final figures were assembled using Adobe Illustrator 2023 (27.4.1; Adobe, San Jose, CA, USA). All raw data are provided as supplemental material.

## RESULTS

### RH shock affects mortality and bloodfeeding in *Ae. aegypti*

In a pilot experiment, mosquitoes were exposed to RH shock treatments for 6, 12, and 18 hours of exposure (HE) to find the exposure levels that would allow mosquitoes to live in sufficient numbers to complete the experiments. We detected 5% mortality under 35% RH conditions at 18 HE (Fig. S1), contrasting with the complete lack of mortality observed in the 75% RH and control (standard insectary RH; 80% RH) treatments. When mosquitoes were exposed for >18 hours, mortality significantly differed between treatments, with mosquitoes dying at higher rates in the 35% RH (48%) compared to 2.5% in the 75% RH and 0% in the control treatment (Fig. S1). Since vector competence experiments required mosquitoes to survive for up to 14 days after being exposed to dehydration and virus infection, we chose 18 HE as the time of exposure to RH shock for the rest of the study.

To assess the interaction between RH shock and mosquito biology, we equally distributed a total of 1,065 mosquitoes into the three humidity treatments for 18 HE and measured mortality after exposure (Table 1). We found that after the RH shock treatments, mortality reached 4.5% in the 35% RH treatment, significantly higher than 1.1% and 0.2% in the 75% RH and control treatments, respectively (Table 1). Then, we challenged surviving mosquitoes with MAYV-spiked blood, sorted bloodfed females, and calculated blood-feeding rates based on the proportion of mosquitoes that were observed to be engorged with blood over the total. We observed that the blood-feeding rate was significantly higher in the 75% RH treatment (Table 1).

**TABLE 1:** Mortality and blood-feeding rates of naive mosquitoes exposed to 18 hours of RH that induce dehydration stress

Mortality					Bloodfeeding			
Treatment	Dead	Alive	Rate (%)	*P*-value	Bloodfed	Non-bloodfed	Rate (%)	*P*-value
Control	1	352	0.28	9.33E-05	314	38	89.2	0.007
75% RH	4	354	1.12		336	18	94.92**^*a*^**	
35% RH	16	338	4.52**^*a*^**		302	36	89.35	

^a^ Indicates treatments that significantly differ from the other two. *P*-values were calculated with Fisher’s exact test followed by multiple comparison analyses with Bonferroni correction.

### Dehydration stress does not affect long-term vector competence

**FIG 2:**
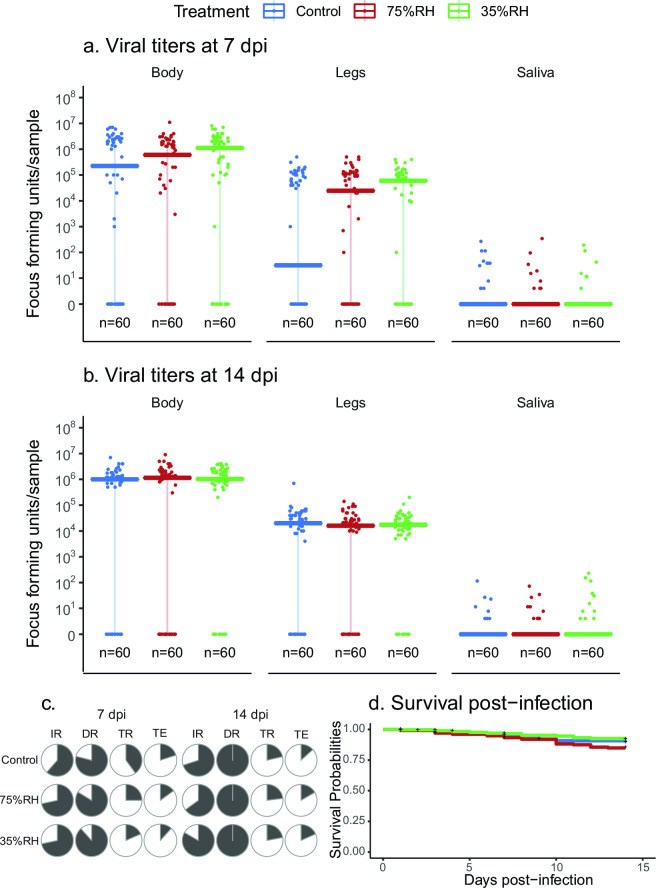
Vector competence at 7 and 14 dpi and survival curve. Viral titers in mosquitoes’ body, legs, and saliva at 7 (a) and 14 dpi (b). n denotes sample size, bars represent the median, and error bars represent data between the first and third quartiles. Virus concentration is presented on a logarithmic scale. (c) Pie charts indicate infection (IR), dissemination (DR), transmission rates (TR), and transmission efficiency (TE). (d) Daily survival probabilities of *Aedes aegypti* after being exposed to dehydration and challenged with MAYV; + indicates when data were censored. We did not detect any statistically significant difference between treatments through the analyses (Kruskal-Wallis, Fisher’s exact test, and log-rank test *P*-value > 0.05).

To understand if RH shock alters the long-term survival and vector competence of mosquitoes challenged with viruses, we returned mosquitoes to standard insectary conditions, and vector competence was evaluated by dissecting relevant tissues at 7 and 14 dpi. When we compared viral titers of the body, legs, and saliva, they were similar between RH treatments and replicates in all three tissues, showing no significant difference for either time point (Fig. 2a and b). Our results show that there is no difference in the IR, DR, TR, and TE between treatments at both tested time points (Fig. 2c). Daily mosquito deaths were counted, and we used survival curves to assess differences between treatments. We found that there is no significant difference between the survival of mosquitoes for each RH shock treatment (Fig. 2d).

### RH shock prompts mortality in infected mosquitoes without affecting short-term viral infection

Since we did not observe altered vector competence due to the long-term effects of RH shock, we aimed to understand the short-term effects of virus infection in mosquitoes that were first infected with MAYV and then suffered RH shock (Fig. 1b). We challenged mosquitoes with MAYV, held them under standard insectary conditions, and then exposed them for 18 HE to the same three RH treatments at 5 and 12 dpi. These two time points were used because of viral competence collection time points at 7 and 14 dpi. Mortality rates were immediately recorded after the 18 HE (at 6 and 13 dpi). Our results (Table 2) show that mortality increases in the 35% RH treatment at 6 dpi, reaching a mortality of 42% that significantly differed from the 20% in the control treatment. Such difference between treatments is also found at 13 dpi, when the mortality in the 35% RH treatment (47%) is significantly higher than the control (22%).

**TABLE 2:** Mortality rates of mosquitoes exposed to 18 hours of dehydration stress at 6 and 13 dpi

Mortality									
6 dpi					13 dpi				
Treatment	Dead	Alive	Rate (%)	*P*-value	Treatment	Dead	Alive	Rate (%)	*P*-value
Control	14	57	19.71	0.01	Control	14	50	21.88	4.42e-05
75% RH	22	50	30.55		75% RH	7	52	11.86	
35% RH	30	41	42.25^*a*^^,^^*b*^		35% RH	30	34	46.88^*a*^	

^*a*^ Indicates the treatment that significantly differs from the other two.

^*b*^ Indicates that the treatment significantly differs from the control. *P*-values were calculated with Fisher's exact test, followed by multiple comparison analyses with Bonferroni correction.

**FIG 3:**
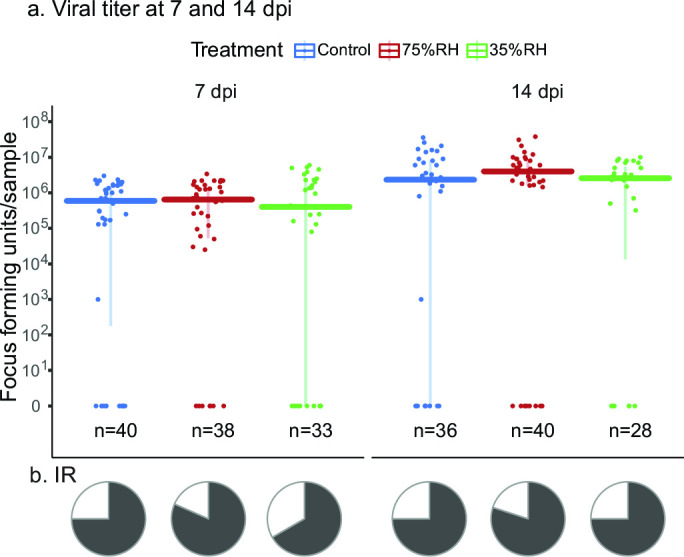
Vector competence of mosquitoes exposed to humidity treatments after being challenged with MAYV. (a) Data are shown as viral titers in mosquitoes’ bodies at 7dpi and 14 dpi. n denotes sample size, bars represent the median, and error bars represent data between the first and third quartiles. Data are shown on logarithmic scale. (b) Pie charts indicate the infection rate (IR). We did not detect a statistically significant difference through the respective statistical analyses (Kruskal-Wallis and Fisher exact test, *P*-value > 0.05).

Once mortality was assessed, mosquitoes were allowed to recover for 24 hours under standard insectary conditions so the virus could resume replication. In this experiment, we measured whole-body viral titers to understand the short-term effect of RH shock on MAYV infection. Results are reported as viral titers in the bodies (Fig. 3a) and prevalence of infection (IR, Fig. 3b). Our results indicate that exposure to varying RH after viral challenge did not affect the IR nor the viral titers at either time point.

## DISCUSSION

Dehydration in mosquitoes occurs due to a combination of lack of access to water, increases in temperature, and decreases in environmental humidity (7). Mosquitoes can use multiple strategies to counter dehydration, such as resting in microhabitats with higher moisture, altering their activity patterns, and increasing blood-feeding activity [reviewed in reference (8)]. Under our experimental design, mosquitoes were exposed to dehydration through RH shock treatments, without the possibility of using such strategies to curtail dehydration during exposure to RH shock. We found that dehydrating *Ae. aegypti* mosquitoes at 75% RH is enough to increase blood-feeding rates without compromising mortality, while exposure to low RH increased mortality, consistent with previous observations in *Culex pipiens* in which dehydration increased blood-feeding behavior but severed dehydration boosted mortality (7). These mortality effects were increased when mosquitoes were previously infected with MAYV, suggesting that viral infection may increase the sensitivity to dehydration stress in mosquitoes. This has been previously shown with other arboviruses, and stressors originated from environmental variables such as thermal stress (35–37), likely due to the demand of resources for maintaining cellular homeostasis, the cost of immune responses against the viral infection, and virus-mediated changes in gene expression (38–40). Importantly, dehydration can be extremely stressful and require specific factors to maintain cellular homeostasis and allow recovery that could very well be impaired during an active viral infection (7, 22, 28). In addition, we found that once mosquitoes survived dehydration and were placed under standard insectary conditions, they showed no difference in daily survival between treatments for a period of 14 days (Fig. 2d), supporting the theory that bloodfeeding allows mosquitoes to recover from dehydration (41) even when facing a newly acquired viral infection.

In this study, we tested the long-term effects of dehydration stress on viral vector competence and the short-term effects of viral infection. In our case, we did not observe a change in IR, DR, TR, and TE when mosquitoes faced different levels of dehydration for a period of 18 HE (Fig. 2a through c ; Fig. 3). There is a large body of literature showing that viral vector competence can be impaired under circumstances that stress the mosquito, including changes in environmental variables such as temperature (2, 29–31). Since dehydration induces physiological changes in the mosquito (22, 28, 42–44), we hypothesized that dehydration would affect vector competence as well. However, it is possible that periods longer than 18 HE to low humidity conditions are required for dehydrating mosquitoes enough to affect their viral vector competence, which could be challenging considering the increase in mortality reported here (Fig. S1; Table 1). Repeated bouts of dehydration have been shown to directly impact mosquito physiology and reproduction (44), suggesting that future studies may want to target how chronic and repeated bouts of exposure to RH stress may impact viral transmission. Although dehydration did not affect vector competence in these experiments, it does not necessarily mean that dehydration does not affect transmission dynamics. Since mortality and density of the mosquitoes (vectors) affect arbovirus transmission (45, 46), it could be considered that a period of dehydration will influence the transmission dynamics of arboviruses by impacting other biological factors such as mortality and feeding rates, as previously modeled for *Culex pipiens* and West Nile virus (7).

MAYV is primarily transmitted by arboreal *Haemagogus* mosquitoes from non-human primates to humans in a sylvatic cycle (47), but maintenance of this mosquito species is challenging under laboratory conditions (48) and hinders vector-pathogen interaction studies. There is evidence of natural infection of MAYV in adult *Ae. aegypti* from Brazil (49), which is a competent vector of MAYV under laboratory conditions. Thus, *Ae. aegypti* stands as an important model species for the study of vector-MAYV interactions under laboratory conditions.

Several aspects that were not covered in this research should be considered for future studies. First, *Ae. aegypti* is present worldwide (15), and environmental variables such as RH, precipitation, and temperature differ between locations and habitats. Mosquitoes distributed in dryer areas have been found to change their phenotype to decrease water loss (25, 26), so studying *Ae. aegypti* strains derived from different mesoclimates could be informative. As vectorial capacity relies heavily on the extrinsic incubation period (EIP) of the virus inside the mosquito (46), it will be relevant to assess how dehydration and RH might affect the length of the EIP in further studies of vector competence. Recent comparative studies between *Ae. aegypti* populations have shown differences in feeding patterns and viral transmission in relation to environmental factors and urbanization (50, 51), which suggests that dehydration and viral transmission dynamics may vary between *Ae. aegypti* lineages. MAYV has been shown to infect some species of *Anopheles* mosquitoes *in vivo* and *in vitro* (32, 52, 53). Several *Anopheles* species (that are also vectors of malaria) are distributed in the Americas [reviewed in reference (54)], including countries where MAYV has been reported, such as Colombia, Venezuela, and Brazil (55). Although some progress has been made to decipher how RH affects the biology of these mosquitoes (56, 57), more research is still required. Thus, it would be important to explore how RH affects the mortality and vector-MAYV interactions in *Anopheles*.

In conclusion, our work suggests that dehydration shock can increase blood-feeding behavior and mortality in mosquitoes, depending on the severity of dehydration. However, mosquitoes are able to recover from this state once RH increases to more stable levels and food sources become available. In addition, under this experimental design, we found that dehydration induced by RH shock did not play a role in driving variation in viral vector competence in *Ae. aegypti*. Finally, we suggest that further studies should explore the relationship between RH and vector competence (including EIP) in several viral strains and species of mosquitoes that are competent vectors of MAYV.

## Data Availability

All data are available as supplemental material.

## SUPPLEMENTAL MATERIAL

Data Set S1 jvi.00695-23-s0001.xlsx Experiment A: Mortality and blood-feeding data.

Data Set S2 jvi.00695-23-s0002.xlsx Experiment A: Survival data.

Data Set S3 jvi.00695-23-s0003.xlsx Experiment A: Viral titer and infection data.

Data Set S4 jvi.00695-23-s0004.xlsx Experiment B: Mortality data.

Data Set S5 jvi.00695-23-s0005.xlsx Experiment B: Viral titer and infection data.

Supplemental material jvi.00695-23-s0006.docx Supplemental figures and data.

ASM does not own the copyrights to Supplemental Material that may be linked to, or accessed through, an article. The authors have granted ASM a non-exclusive, world-wide license to publish the Supplemental Material files. Please contact the corresponding author directly for reuse.
