# Supplementary material for: Dehydration stress and Mayaro virus vector competence in Aedes aegypti
Source: J Virol. 2023 Dec 5;97(12):e00695-23. doi: 10.1128/jvi.00695-23 (PMC10734514; doi:10.1128/jvi.00695-23)
Supplement: Supplemental material — Supplemental figures and data. [file jvi.00695-23-s0006.docx]

**Supplemental material**

**Methods**

*Assessment of mortality at different timepoints of exposure to relative humidity treatments*

Three humidity treatments were prepared: 75% RH, 32%RH, and control treatment, the latter was set at regular insectary humidity conditions (80%RH). To reach 75% and 35% relative humidity conditions, chambers were crafted with plastic transparent containers, holding cups filled with supersaturated solutions of NaCl and MgCl_2_ in the inside respectively. Three- to five-days old female mosquitoes were anesthetized with ice and sorted into nine 20x30x20 board cages in groups of 120 individuals, then held for a day at normal insectary conditions to allow them to recover. The next day, mosquitoes were deprived of access to water, and cages were equally divided between the three humidity treatments. Mortality was recorded for all the cages every 6 hours, and one cage per treatment was selected for immediately feeding on human blood for 1 hour to compare blood-feeding rates. The cages that were selected in each timepoint were then excluded from the rest of the experiment. Two replicates were performed, the timepoints for each replicate were 6, 12 and 18; and 12, 18 and 24 hours of exposure (HE) respectively.

**Results**

**Table S1. Viral tiers of aliquots of infectious blood offered to mosquitoes**. Samples were stored in cold at -80°C, until virus titering with FFA. Results differ from initial concentration (1E+07) because freezing and thawing the samples can decrease their viral loads. For experimental details see figure 1, and methods section of the paper.

| FFAs of infectious blood meal | | | |
| --- | --- | --- | --- |
| Experiment | Replicate | Viral titer in blood | Average |
| Long-term (a) | 1 | 5.60E+06 | 6.07E+06 |
|  | 2 | 7.00E+06 |  |
|  | 3 | 5.60E+06 |  |
| Short-term (b) | 1 | 7.75E+06 | 6.04E+06 |
|  | 2 | 4.33E+06 |  |


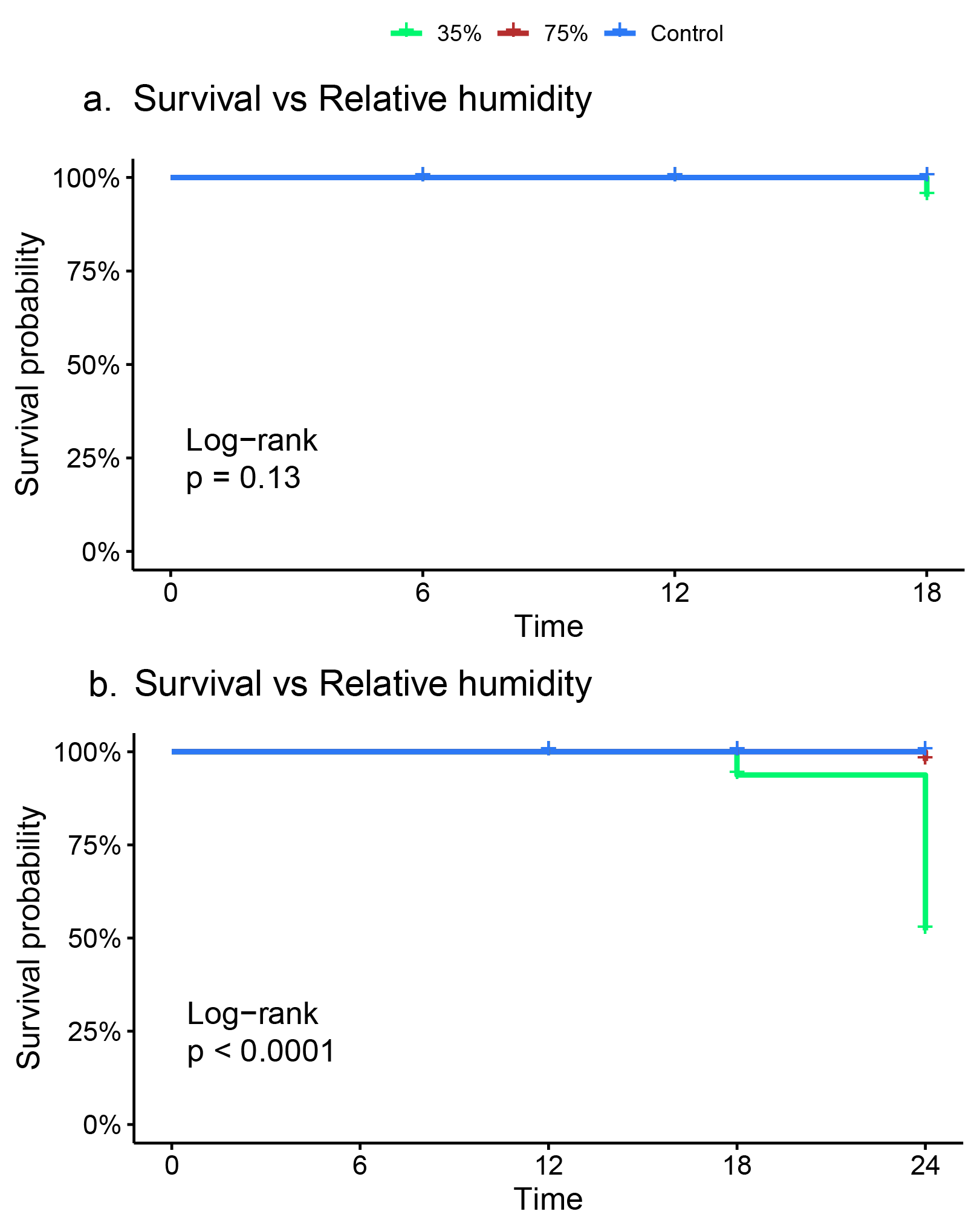


**Figure S1. Mosquito mortality per HE to the humidity treatments.** Each graph represents the survival curve of mosquitoes challenged with the temperature treatments for different times of exposure in 2 replicates. **a.** The first replicate tested the timepoints 6, 12 and 18 HE. **b.** Second replicate tested 12, 18 and 24 HE timepoints. Results are presented in the “RH shock affects mortality and bloodfeeding in Ae. Aegypti” section of the paper results.

**Supplemental datasets**

Supplemental Data Set S1: Experiment A Mortality and bloodfeeding data.

Supplemental Data Set S2: Experiment A survival data

Supplemental Data Set S3: Experiment A viral titer and infection data

Supplemental Data Set S4: Experiment B Mortality and bloodfeeding data.

Supplemental Data Set S5: Experiment B viral titer and infection data
